# Acute and longitudinal magnetic resonance imaging abnormalities in antibody-mediated encephalitis

**DOI:** 10.1093/braincomms/fcag193

**Published:** 2026-06-17

**Authors:** Nabil Seery, Paul Beech, Robb Wesselingh, Mark Schoenwaelder, James Broadley, Laurie McLaughlin, Tiffany Rushen, Liora ter Horst, Andrew Duncan, Tracie Tan, Christina Kazzi, Genevieve Skinner, Cassie Nesbitt, Katherine Buzzard, Wendyl J D’Souza, Yang Tran, Anneke van der Walt, Amy Halliday, Mirasol Forcadela, Bruce Taylor, Andrew Swayne, Amy Brodtmann, David Gillis, Ernest G Butler, Tomas Kalincik, Udaya Seneviratne, Richard A Macdonell, Sudarshini Ramanathan, Stefan Blum, Charles B Malpas, Stephen W Reddel, Todd A Hardy, Terence J O’Brien, Paul Sanfilippo, Helmut Butzkueven, Mastura Monif, Australian Autoimmune Encephalitis Consortium, Mastura Monif, Amy Brodtmann, Amy Halliday, Bruce Taylor, Cassie Nesbitt, Catherine Meade, Charles B Malpas, Chris Kyndt, Christina Kazzi, David Gillis, David Tarlinton, Darshi Ramanathan, Ernie G Butler, Foong Yi, Genevieve Skinner, Hannah Ford, Helmut Butzkueven, James Beharry, Jayashri Kulkarni, Jenny MacIntyre, Joshua Yap, Joanne Fielding, Kath Buzzard, Katherine Ko, Laurie McLaughlin, Marie O’Shea, Meaghan Clough, Meng Tan, Miri Forcadel, Nabil Seery, Nicola Warren, Noushin Chini Foroush, Owen White, Paul Beech, Richard A Macdonell, Richard Wong, Robb Wesselingh, Rubina Alpitsis, Robert Bourke, Simon Broadley, Stefan Blum, Stephen W Reddel, Terence J O’Brien, Tiffany Rushen, Todd A Hardy, Tomas Kalincik, Tracie Tan, Udaya Seneviratne, Wen Wen Zhang, Wendyl J D’Souza

**Affiliations:** Department of Neuroscience, School of Translational Medicine, Monash University, Melbourne, Victoria 3004, Australia; Department of Neurology, Alfred Health, Melbourne, Victoria 3004, Australia; Department of Radiology, Alfred Health, Melbourne, Victoria 3004, Australia; Department of Radiology, Monash Health, Clayton, Victoria 3168, Australia; Department of Neuroscience, School of Translational Medicine, Monash University, Melbourne, Victoria 3004, Australia; Department of Neurology, Alfred Health, Melbourne, Victoria 3004, Australia; Department of Radiology, Alfred Health, Melbourne, Victoria 3004, Australia; Department of Neuroscience, School of Translational Medicine, Monash University, Melbourne, Victoria 3004, Australia; Department of Neurology, Alfred Health, Melbourne, Victoria 3004, Australia; Department of Neurology, Princess Alexandra Hospital, Woolloongabba, Queensland 4102, Australia; School of Medicine, The University of Queensland, UQ, Herston, Queensland 4006, Australia; Department of Neuroscience, School of Translational Medicine, Monash University, Melbourne, Victoria 3004, Australia; Department of Neurology, Alfred Health, Melbourne, Victoria 3004, Australia; Department of Neurology, Princess Alexandra Hospital, Woolloongabba, Queensland 4102, Australia; School of Medicine, The University of Queensland, UQ, Herston, Queensland 4006, Australia; Department of Medicine, St Vincent’s Hospital, University of Melbourne, Fitzroy, Victoria 3065, Australia; Department of Neuroscience, School of Translational Medicine, Monash University, Melbourne, Victoria 3004, Australia; Department of Neurology, Alfred Health, Melbourne, Victoria 3004, Australia; Department of Neuroscience, School of Translational Medicine, Monash University, Melbourne, Victoria 3004, Australia; Department of Neurology, Alfred Health, Melbourne, Victoria 3004, Australia; Department of Neurology, Princess Alexandra Hospital, Woolloongabba, Queensland 4102, Australia; School of Medicine, The University of Queensland, UQ, Herston, Queensland 4006, Australia; Department of Neurology, Alfred Health, Melbourne, Victoria 3004, Australia; Department of Neuroscience, Barwon Health, Geelong, Victoria 3220, Australia; Department of Neurosciences, Eastern Health, Box Hill, Victoria 3128, Australia; Department of Medicine, St Vincent’s Hospital, University of Melbourne, Fitzroy, Victoria 3065, Australia; Department of Medicine, St Vincent’s Hospital, University of Melbourne, Fitzroy, Victoria 3065, Australia; Department of Pathology, St Vincent’s Hospital, Fitzroy, Victoria 3065, Australia; Department of Neuroscience, School of Translational Medicine, Monash University, Melbourne, Victoria 3004, Australia; Department of Neurology, Alfred Health, Melbourne, Victoria 3004, Australia; Department of Medicine, St Vincent’s Hospital, University of Melbourne, Fitzroy, Victoria 3065, Australia; Department of Neurology, Austin Health, Heidelberg, Victoria 3084, Australia; Department of Neurology, Royal Hobart Hospital, Hobart, Tasmania 7000, Australia; Department of Neurology, Princess Alexandra Hospital, Woolloongabba, Queensland 4102, Australia; School of Medicine, The University of Queensland, UQ, Herston, Queensland 4006, Australia; Department of Neuroscience, School of Translational Medicine, Monash University, Melbourne, Victoria 3004, Australia; Department of Neurosciences, Eastern Health, Box Hill, Victoria 3128, Australia; Department of Neurology, Royal Melbourne Hospital, Parkville, Victoria 3052, Australia; Division of Immunology, Pathology Queensland Central Laboratory, Herston, Queensland 4006, Australia; Department of Neurology, Peninsula Health, Frankston, Victoria 3199, Australia; Department of Neurology, Royal Melbourne Hospital, Parkville, Victoria 3052, Australia; Department of Medicine, CORE, The University of Melbourne, Parkville, Victoria 3010, Australia; Department of Neurology, Neuroimmunology Centre, Royal Melbourne Hospital, Melbourne, Victoria 3000, Australia; Department of Neuroscience, School of Translational Medicine, Monash University, Melbourne, Victoria 3004, Australia; Department of Neurology, Austin Health, Heidelberg, Victoria 3084, Australia; Department of Neuroscience, Monash Health, Clayton, Victoria 3168, Australia; Department of Neurology, Austin Health, Heidelberg, Victoria 3084, Australia; Department of Neurology and Concord Clinical School, Concord Hospital, Concord, New South Wales 2139, Australia; Translational Neuroimmunology Group, Kids Neuroscience Centre and Brain and Mind Centre, Faculty of Medicine and Health, University of Sydney, Westmead, New South Wales 2145, Australia; Department of Neurology, Princess Alexandra Hospital, Woolloongabba, Queensland 4102, Australia; School of Medicine, The University of Queensland, UQ, Herston, Queensland 4006, Australia; Department of Neuroscience, School of Translational Medicine, Monash University, Melbourne, Victoria 3004, Australia; Department of Medicine (Royal Melbourne Hospital), University of Melbourne, Parkville, Victoria 3052, Australia; Melbourne School of Psychological Sciences, University of Melbourne, Parkville, Victoria 3052, Australia; Department of Neurology and Concord Clinical School, Concord Hospital, Concord, New South Wales 2139, Australia; Brain and Mind Centre, University of Sydney, Camperdown, New South Wales 2050, Australia; Department of Neurology and Concord Clinical School, Concord Hospital, Concord, New South Wales 2139, Australia; Brain and Mind Centre, University of Sydney, Camperdown, New South Wales 2050, Australia; Department of Neuroscience, School of Translational Medicine, Monash University, Melbourne, Victoria 3004, Australia; Department of Neurology, Alfred Health, Melbourne, Victoria 3004, Australia; Department of Neuroscience, School of Translational Medicine, Monash University, Melbourne, Victoria 3004, Australia; Department of Neuroscience, School of Translational Medicine, Monash University, Melbourne, Victoria 3004, Australia; Department of Neurology, Alfred Health, Melbourne, Victoria 3004, Australia; Department of Neuroscience, School of Translational Medicine, Monash University, Melbourne, Victoria 3004, Australia; Department of Neurology, Alfred Health, Melbourne, Victoria 3004, Australia

**Keywords:** MRI, LGI1, NMDAR, hippocampal swelling

## Abstract

Brain magnetic resonance imaging (MRI) abnormalities are an important finding in the evaluation of patients with suspected autoimmune encephalitis (AE). There have been few studies evaluating the frequency and prognostic significance of MRI abnormalities, especially hippocampal swelling, in anti-*N*-methyl-D-aspartate receptor (NMDAR) and anti-leucine-rich glioma-inactivated 1 (LGI1) Ab-mediated encephalitides. We conducted a multi-centre, retrospective study involving adult patients with confirmed antibody-mediated encephalitis and at least one MRI scan from 10 Australian hospitals (*n* = 139). MRI scans were evaluated by a neuroradiologist blinded to the specific autoimmune encephalitis diagnosis. We evaluated associations between acute MRI abnormalities (e.g. hippocampal swelling) with 12-month function (modified Rankin scale, mRS ≥2 = worse outcome) and radiological findings. In patients with anti-LGI1 Ab-mediated encephalitis, we identified hippocampal swelling on initial MRI to be associated with worse function at 12 months (OR 0.03; 95% CI 0.003, 0.34; *P* = 0.005). We found initial AE-associated T2/fluid attenuated inversion recovery (FLAIR) hyperintensities were not associated with 12-month mRS in either the anti-NMDAR (OR 0.39; 95% CI 0.04, 3.97; *P* = 0.42) or anti-LGI1 Ab-mediated encephalitis groups (OR 0.34; 95% CI 0.07, 1.57; *P* = 0.17). In anti-LGI1 Ab-mediated encephalitis, both hippocampal swelling (OR 5.76; 95% CI 1.14, 29.02; *P* = 0.03) and T2/FLAIR hyperintensity (OR 6.81; 95% CI 1.28, 36.22; *P* = 0.03) were related to the development of mesial temporal atrophy and hippocampal sclerosis. Acute hippocampal swelling is associated with worse outcomes in anti-LGI1 Ab-mediated encephalitis and, alongside initial T2/FLAIR hyperintensity, is associated with the development of both mesial temporal atrophy and hippocampal sclerosis.

## Teaser

Seery *et al*. report that in anti-leucine-rich glioma-inactivated 1 encephalitis patients, hippocampal swelling detected on initial MRI is associated with worse functional capacity at 12 months after disease onset, and future development of hippocampal atrophy and sclerosis, as assessed by visual inspection by a neuroradiologist blinded to the underlying diagnosis.

## Graphical Abstract

**Graphical Abstract:**
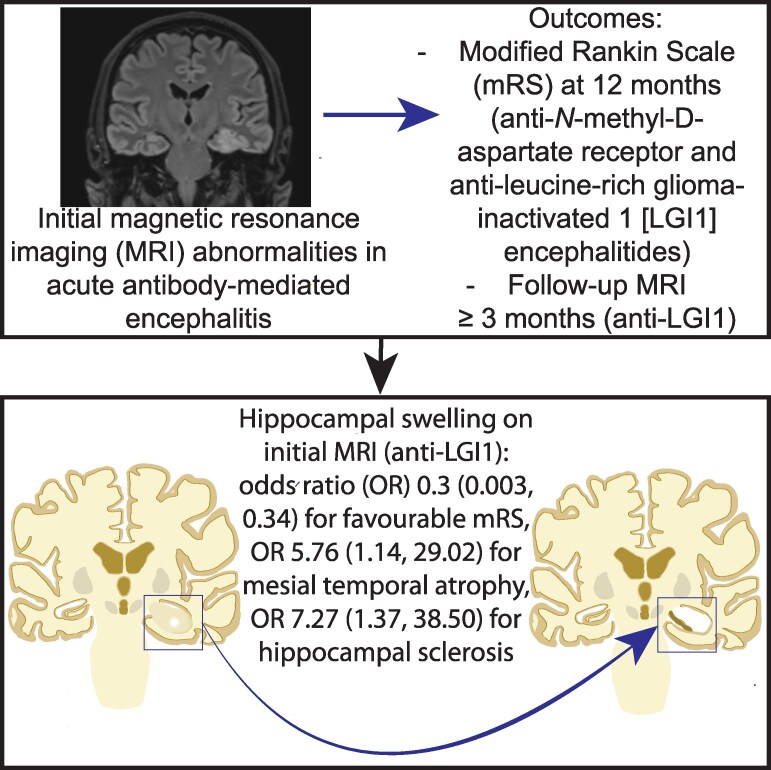

## Introduction

Antibody (Ab)-mediated encephalitides comprise most autoimmune encephalitis (AE) subtypes.^1^ Of these, anti-*N*-methyl-D-aspartate receptor (NMDAR) and anti-leucine-rich glioma-inactivated 1 (LGI1) Ab-mediated encephalitides are the most common.^2^ Prompt diagnosis and early commencement of immunotherapy are associated with better outcomes across common Ab-mediated subtypes.^3-5^ Antibody results are often delayed in the acute setting, such that paraclinical investigations, particularly magnetic resonance imaging (MRI) and cerebrospinal fluid (CSF) evaluation, commonly guide initial treatment decisions. MRI is used both to exclude relevant differential diagnoses, but can also support a likely AE diagnosis.^6^ Misdiagnosis is an increasingly recognized problem^7^ that may expose a patient to undue risks of immunosuppression, or potentially delay treatment for an alternative underlying disease. Neurologic, psychiatric, infectious, toxic and neoplastic conditions are in the differential diagnosis and can mimic abnormalities seen in AE.^8^ Therefore, accurate and early MRI interpretation is essential in AE diagnosis.

MRI findings can also prognosticate certain forms of AE^9,10^ and potentially guide immunotherapy use. However, the significance of MRI abnormalities may vary with the underlying disease. MRI abnormalities are more frequently seen in patients with anti-LGI1 Ab-mediated encephalitis (LGI1 patients) than in anti-NMDAR Ab-mediated encephalitis (NMDAR patients).^2^ Despite this, few studies have evaluated the prognostic utility of routine MRI abnormalities in LGI1 patients.^11^ In NMDAR patients, encephalitis-suggestive MRI abnormalities has been variably associated with worse outcomes, which warrants further clarification.^9,12^

We evaluated acute and longitudinal MRI abnormalities in patients with Ab-mediated encephalitis and explored their prognostic significance in NMDAR and LGI1 patients.

## Materials and methods

This study was approved by the central Human Research Ethics Committee at Alfred Health (HREC/17/Alfred/168), with a waiver of consent for medical record access for retrospectively recruited cases. All prospectively recruited cases provided informed consent.

### Participant identification and inclusion criteria

Patients aged 18 and over with confirmed Ab-mediated encephalitis and available brain MRI scans were identified from 10 hospitals as part of the Australian Autoimmune Encephalitis Consortium, between January 2008 and August 2024. NMDAR patients were included if they met the Lancet Neurology AE diagnostic criteria.^13^ In the context of a compatible clinical syndrome, other Ab-mediated encephalitides were diagnosed if a serum or CSF antibody was detected using a cell-based assay.

### Data collection

**Figure 1:**
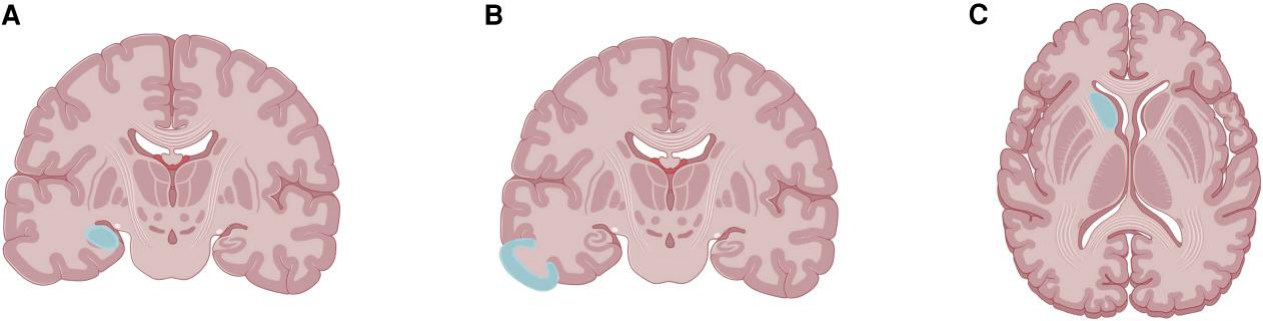
**Schematic depiction of anatomical categorization of T2/FLAIR hyperintensities used in this study.** (**A**) mesial temporal, (**B**) extra-mesial temporal, (**C**) extra-temporal. FLAIR, fluid-attenuated inversion recovery. ‘Created in BioRender. Monif, M. (2026) https://BioRender.com/t8vxsy6’.

Clinical data was collected retrospectively from medical records, or prospectively during clinic visits. We recorded date of symptom onset, immunotherapies and MRI scans and clinical features observed during admission or first outpatient visit. Disability was assessed using the modified Rankin Scale (mRS)^14^ and clinical assessment scale in autoimmune encephalitis^15^ (CASE). The CASE scale is a 9-item AE severity clinical scale. Serial clinically available MRI scans for each patient were analysed by a neuroradiologist (P.B.) blinded to the underlying diagnosis. As a minimum, scan protocols included T1, T2, FLAIR, DWI and ADC sequences. Most initial scans had a post-gadolinium T1 sequence. The presence and anatomical location of T2/FLAIR hyperintensity were recorded for each scan, classified as mesial temporal if within the hippocampi or amygdala, temporal if in the temporal lobes outside of the mesial temporal structures, and extra-temporal if present in any other supratentorial or infratentorial brain region (Fig. 1). The presence of diffusion restriction, T1 post-contrast gadolinium enhancement, hippocampal swelling, focal atrophy and hippocampal sclerosis were also assessed serially. Focal atrophy was recorded as mesial temporal, temporal, frontal, parietal, occipital or cerebellar in location. Additionally, for the first 43 patients with available imaging in a central neuroarchiving system, the initial, and where present, final MRI scan were analysed by a second blinded neuroradiologist (M.S) using the same framework, to assess inter-rater agreement. Clinical data were recorded using the Research Electronic Data Capture (REDCap) database.^16,17^

### Study definitions and design

T2/FLAIR hyperintensity on MRI was considered abnormal and related to AE if no better cause, such as stroke, demyelination, chronic small vessel ischaemia or post-surgical change was deemed a more likely explanation. Abnormalities unrelated to AE were not included in analyses. Diffusion restriction was recorded as hyperintensity in any given brain region on diffusion-weighted imaging (DWI), if accompanied by hypointensity in the equivalent location on the apparent diffusion coefficient sequence. Hippocampal swelling was recorded at neuroradiologist discretion by evaluating apparent size in comparison to surrounding structures, including the amgydala and CSF. Contrast-enhanced lesions (CEL) represented parenchymal lesions hyperintense on T1 gadolinium-enhanced MRI sequences but not hyperintense on corresponding unenhanced T1 sequences. Hippocampal sclerosis was defined if both hyperintensity and atrophy were present in the same hippocampus.^18,19^ For MRI changes over time, follow-up MRI to evaluate regional volume changes had to be performed at least 3 months following the initial MRI, to allow for evolution of atrophy.^20^ T2/FLAIR hyperintensity persistence was evaluated on an MRI at least 1 month from the initial MRI to allow seizure-related changes to resolve.^21^

A ‘favourable’ mRS score was ≤2. Nadir mRS was recorded at any time point prior to the first of 12 months following an initial clinical encounter (e.g. hospital admission or initial outpatient clinic appointment) or first clinical relapse. We defined ‘significant’ memory impairment as a score of ≥2 in the memory component of the CASE. First-line immunotherapy included pulsed methylprednisolone, induction intravenous immunoglobulin and plasma exchange. Time to first-line immunotherapy was measured as the time from the time of symptom onset until commencement of first-line immunotherapy. To prevent bias due to exclusion of untreated cases, time to first-line immunotherapy was assigned as time from symptom onset to outcome in relevant analyses, and cases who died prior to 12 months due to their condition or related complications were assigned an mRS of 6.

### Statistical analysis

All analyses were performed using R version 4.4.1. Descriptive statistics were reported as median and interquartile range (IQR). Inter-rater concordance was assigned for a given radiological parameter if there was agreement on the presence or absence of a given abnormality on both sides. Each possible combination of right and left sided abnormalities for a given parameter were assigned a score. Inter-rater agreement for these scores were assessed using Gwet’s agreement coefficient (AC1).^22^ We considered agreement to be slight (0 to 0.20), fair (0.21 to 0.40), moderate (0.41 to 0.60), substantial (0.61 to 0.80), or almost perfect (>0.80).^23^ Univariable and multivariable logistic regression models were constructed to evaluate MRI predictors of favourable mRS at 12 months using listwise deletion, in NMDAR and LGI1 patients. Collinearity between covariates was assessed using variance inflation factors. Potential predictor variables with low frequency were not analysed. Separate multivariable models were constructed for the presence of T2/FLAIR hyperintensity, and for any other MRI parameters with *P* < 0.05 in univariable analysis. For multivariable predictors of favourable mRS, the covariates age, time to immunotherapy and nadir mRS were pre-selected, based on previous studies demonstrating time to treatment and measures of initial disease severity associating with worse outcomes in both diseases,^3,4^ and we included any other clinical variables with *P* < 0.05 in univariable analysis. We only performed predictive models with mesial temporal atrophy or hippocampal sclerosis on follow-up MRI as the outcome in LGI1 patients due to the rarity of these outcomes in other subtypes. Separate multivariable models were constructed for MRI predictors with *P* < 0.05 in univariable analysis. We only included time to first-line immunotherapy as a pre-specified covariate in multivariable models, due to the respective sample sizes. Finally, we analysed the relationship of mesial temporal atrophy and hippocampal sclerosis on the last available MRI at least 3 months following the initial scan with significant memory impairment at 12 months in LGI1 patients using multivariable logistic regression. Age at symptom onset was the only pre-selected clinical covariate, again due to the limited number of LGI1 patients with significant memory impairment at 12 months. Related multivariable logistic regression analyses were represented alongside complementary forest plots, with odds ratios represented by circles proportional to the precision of the estimate.

## Results

A total of 137 patients with Ab-mediated encephalitis and at least one available MRI scan were included in the study. These included 64 patients with anti-NMDAR, 56 with anti-LGI1, 10 with anti-contactin-associated protein-like 2 (CASPR2), 5 with anti-α-amino-3-hydroxy-5-methyl-4-isoxazolepropinoic receptor (AMPAR) and 2 with anti-γ-aminobutyric acid B (GABA_B_) Ab-mediated encephalitis (Table 1). The median age of onset was 49 (IQR 27.66) years; 55 (40%) patients were male (Supplementary Table 1). First line immunotherapy was administered in 134 (98%) patients. The median mRS and CASE scores at 12 months were 2 (IQR 1.2) and 2 (IQR 1.3) respectively; a favourable mRS occurred in 88 (77%) patients at 12 months. Acute clinical features are presented in Supplementary Table 1.

**Table 1:** Initial MRI characteristics

Characteristic	Overall, *n* = 137	NMDAR, *n* = 64	LGI1, *n* = 56	CASPR2, *n* = 10	GABAB, *n* = 2	AMPAR, *n* = 5
Time to MRI, median (IQR), days	19 (8,52)	16 (9,39)	25 (6,78)	20 (8139)	18 (13,22)	25 (9,29)
MRI before 1st line immunotherapy	123 (90%)	56 (89%)	52 (93%)	9 (90%)	2 (100%)	4 (80%)
T2/FLAIR hyperintensity	37 (27%)	7 (11%)	24 (43%)	3 (30%)	0 (0%)	3 (60%)
T2/FLAIR mesial temporal	28 (20%)	3 (5%)	19 (34%)	3 (30%)	0 (0%)	3 (60%)
Bilateral T2/FLAIR mesial temporal	7 (5%)	0 (0%)	5 (9%)	1 (10%)	0 (0%)	1 (20%)
T2/FLAIR temporal	3 (2%)	0 (0%)	3 (5%)	0 (0%)	0 (0%)	0 (0%)
T2/FLAIR extratemporal	7 (5%)	4 (6%)	3 (5%)	0 (0%)	0 (0%)	0 (0%)
DWI	3 (2%)	0 (0%)	2 (4%)	0 (0%)	0 (0%)	1 (20%)
CEL	4 (3%)	1 (2%)	2 (4%)	0 (0%)	0 (0%)	1 (20%)
Hippocampi swelling	17 (12%)	1 (2%)	12 (21%)	2 (20%)	0 (0%)	2 (40%)
Bilateral hippocampi swelling	4 (3%)	0 (0%)	3 (5%)	1 (10%)	0 (0%)	0 (0%)
Focal atrophy	2 (1%)	0 (0%)	1 (2%)	0 (0%)	0 (0%)	1 (20%)
Hippocampal sclerosis	0 (0%)	0 (0%)	0 (0%)	0 (0%)	0 (0%)	0 (0%)

AMPAR, α-amino-3-hydroxy-5-methyl-4-isoxazolepropinoic receptor; CASPR2, contactin-associated protein-like 2; CEL, contrast enhancing lesion; DWI, diffusion weighted imaging; FLAIR, fluid attenuated inversion recovery; GABAB, γ-aminobutyric acid B; LGI1, leucine-rich glioma-inactivated 1; MRI, magnetic resonance imaging; NMDAR, N-methyl-D-aspartate receptor.

### Initial MRI findings

The first MRI occurred at a median of 19 (IQR 8.52) days from symptom onset, and preceded or occurred on the day of first-line immunotherapy in 123 (90%) patients (Table 1). T2/FLAIR hyperintensities occurred in 37 (27%) patients. Data for other MRI parameters are presented in Table 1.

In NMDAR patients, T2/FLAIR hyperintensities were present in only 7 (11%) patients. These were mesial temporal in 3 (5%) patients, and extratemporal in 4 (6%) patients. Hippocampal swelling occurred in 1 (2%) patient, and no patients had focal atrophy or hippocampal sclerosis on initial MRI.

In LGI1 patients, 24 (43%) had T2/FLAIR hyperintensities. Unilateral and bilateral mesial temporal hyperintensities occurred in 19 (34%) and 5 (9%) patients respectively. Other involved areas were extra-mesial temporal in 3 (5%) patients and extra-temporal in 3 (5%) patients. Hippocampal swelling occurred in 12 (21%) patients, which was bilateral in 3 (5%). Focal atrophy was present in 1 (2%) patient, but no patients had hippocampal sclerosis on initial MRI.

Diffusion restriction occurred in 3 (2%) patients, and contrast enhancement in 4 (3%). All instances of diffusion restriction and contrast enhancement occurred as part of a T2/FLAIR hyperintense lesion, and resolved at the time of the subsequent scan, which was performed with gadolinium contrast in all cases. Details of cases with CEL and DWI-positive lesions are presented in Supplementary Table 2. All patients with DWI-positive and CEL lesions presented with seizures except for one anti-LGI1 Ab-mediated encephalitis patient with an initial mesial-temporal CEL who presented with dizzy-spells. One anti-LGI1 Ab-mediated encephalitis patient with a DWI-positive lesion presented with status epilepticus (SE).

### Follow-up MRI findings

**Figure 2:**
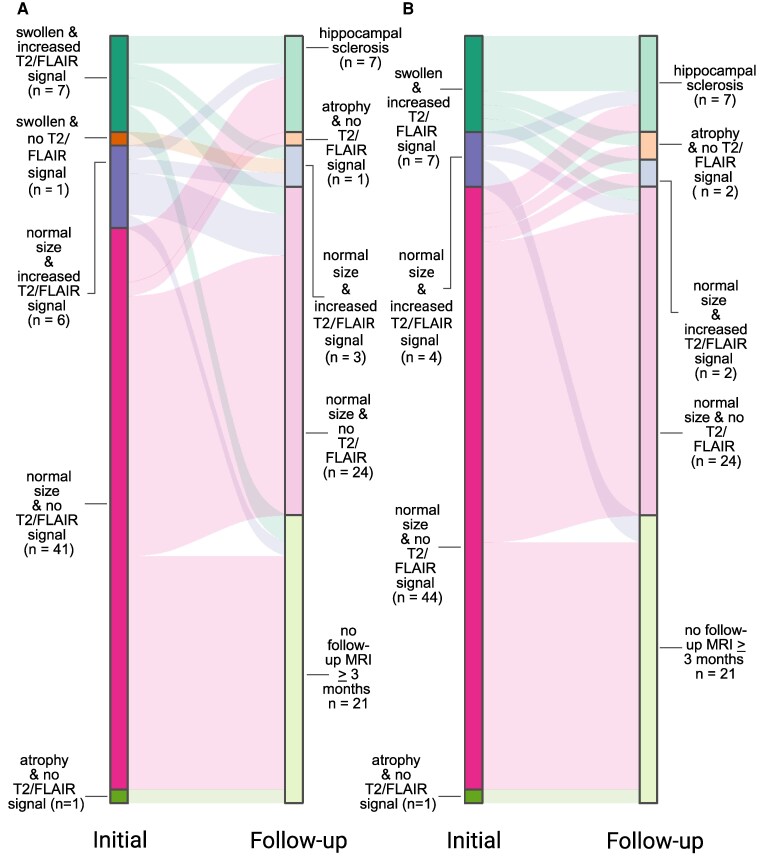
**Sankey diagram demonstrating initial and follow-up MRI findings in LGI1 patients.** Panel (**A**) corresponds to left-sided hippocampus findings, and panel (**B**) right-sided. FLAIR, fluid-attenuated inversion recovery.

In total, 78 patients had a follow-up MRI at least 3 months following the initial MRI, of whom 34 were NMDAR patients and 35 LGI1 patients (Table 2). Focal atrophy occurred in 22 (30%) and mesial temporal atrophy in 21 (27%) patients. Of NMDAR patients, 4 (13%) and 3 (9%) had focal and mesial temporal atrophy, respectively. In LGI1 patients, all atrophy was confined to the mesial temporal region, occurring in 14 (41%) patients. The relationship between initial MRI findings and outcomes on follow-up MRI at least 3 months later in LGI1 patients is presented in Fig. 2. The specific location of atrophy for other AE subtypes is detailed in Table 2. Hippocampal sclerosis occurred in 15 (21%) patients: in 2 (7%) with anti-NMDAR, in 11 (31%) in anti-LGI1 and in 2 (50%) with anti-AMPAR Ab-mediated encephalitis.

**Table 2:** Outcomes in patients with MRI performed at least 3 months from initial MRI

Characteristic	Overall, *N* = 78	NMDAR, *N* = 34	LGI1, *N* = 35	CASPR2, *N* = 4	GABAB, *N* = 1	AMPAR, *N* = 4
Focal atrophy	22 (30%)	4 (13%)	14 (41%)	0 (0%)	1 (100%)	3 (75%)
Mesial temporal atrophy	21 (27%)	3 (9%)	14 (41%)	0 (0%)	1 (100%)	3 (75%)
Temporal atrophy	0 (0%)	0 (0%)	0 (0%)	0 (0%)	0 (0%)	0 (0%)
Frontal atrophy	0 (0%)	0 (0%)	0 (0%)	0 (0%)	0 (0%)	0 (0%)
Parietal atrophy	0 (1.3%)	0 (0%)	0 (0%)	0 (0%)	0 (0%)	0 (0%)
Occipital atrophy	0 (0%)	0 (0%)	0 (0%)	0 (0%)	0 (0%)	0 (0%)
Cerebellar atrophy	1 (1%)	1 (3%)	0 (0%)	0 (0%)	0 (0%)	0 (0%)
Hippocampal sclerosis	15 (21%)	2 (7%)	11 (31%)	0 (0%)	0 (0%)	2 (50%)

AMPAR, α-amino-3-hydroxy-5-methyl-4-isoxazolepropinoic receptor; CASPR2, contactin-associated protein-like 2; GABAB, γ-aminobutyric acid B; LGI1, leucine-rich glioma-inactivated 1; NMDAR, N-methyl-D-aspartate receptor.

**Figure 3:**
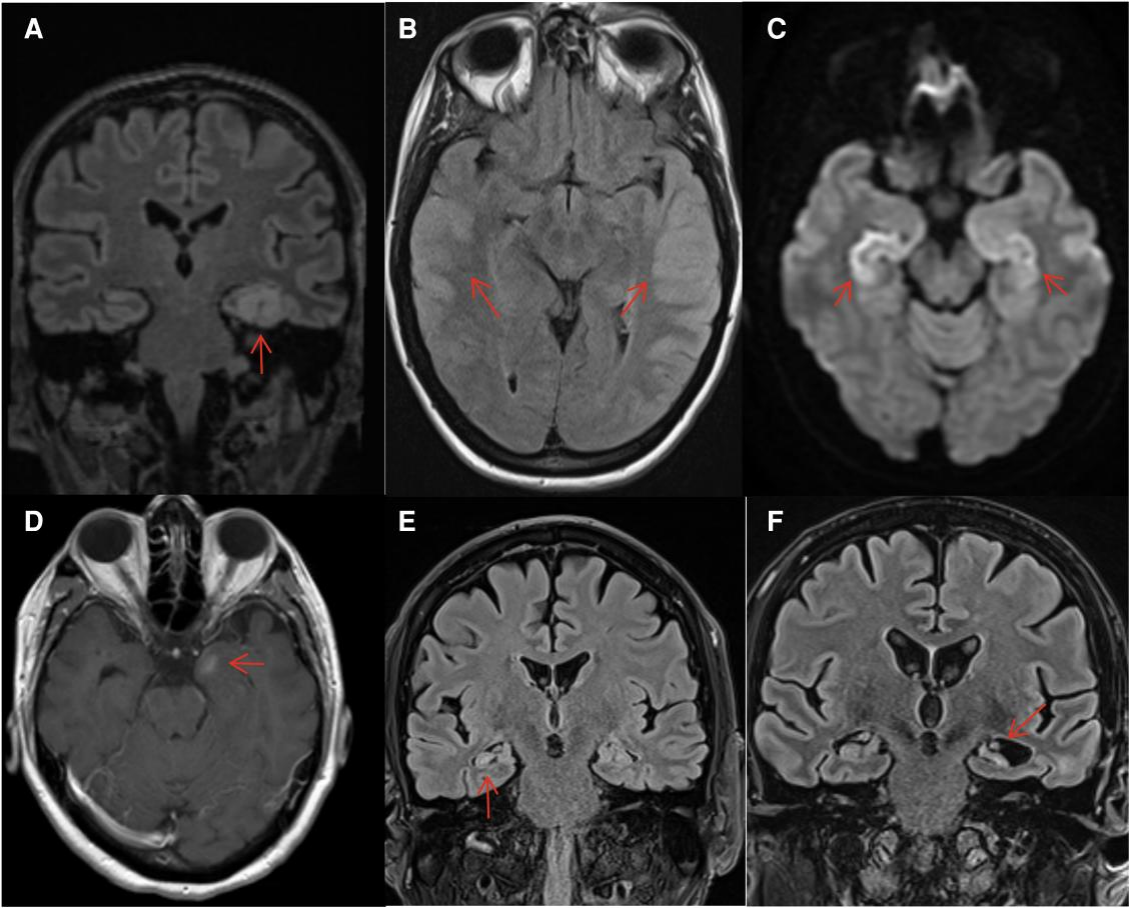
**Representative MRI images with relevant abnormalities.** Abnormalities are indicated by arrows, and demonstrate: (**A**) left sided hippocampal swelling and FLAIR hyperintensity in a patient with anti-LGI1 Ab-mediated encephalitis; (**B**) bilateral temporal FLAIR hyperintensity in a patient with anti-LGI1 Ab-mediated encephalitis; (**C**) bilateral hippocampal diffusion restriction in a patient with anti-LGI1 Ab-mediated encephalitis (associated with ADC reduced signal bilaterally); (**D**) left amygdala T1-weighted contrast enhancement in a patient with anti-AMPAR Ab-mediated encephalitis; (**E**) right sided hippocampal atrophy without sclerosis in a patient with anti-LGI1 Ab-mediated encephalitis; (**F**) left hippocampal sclerosis in a patient with anti-LGI1 Ab-mediated encephalitis. Ab, antibody; ADC, apparent diffusion coefficient; AMPAR, α-amino-3-hydroxy-5-methyl-4-isoxazolepropinoic receptor; FLAIR, fluid-attenuated inversion recovery; LGI1, leucine-rich glioma-inactivated 1.

Of 91 patients with a follow-up MRI at least 1 month after the initial scan, 20 (22%) had T2/FLAIR hyperintensity detected (Table 3). In 10 of 41 patients with anti-LGI1 Ab-mediated encephalitis, T2/FLAIR hyperintensity was present in the mesial temporal lobes without associated atrophy (i.e. no hippocampal sclerosis). Examples of initial and follow-up MRI findings are presented in Fig. 3.

**Table 3:** T2/FLAIR persistence in patients with a follow-up MRI at least 1 month after initial scan

Characteristic	Overall *N* = 91	NMDAR *N* = 39	LGI1 *N* = 41	CASPR2 *N* = 6	GABAB *N* = 1	AMPAR *N* = 4
Time symptoms to MRI	172 (116, 486)	408 (129, 1006)	153 (107, 258)	138 (104, 189)	261 (261, 261)	143 (111, 172)
Time MRI from initial MRI	131 (73, 379)	368 (88, 812)	109 (66, 190)	126 (85, 153)	253 (253, 253)	125 (100, 147)
T2/FLAIR hyperintensity initial MRI	29 (32%)	4 (10%)	20 (49%)	2 (33%)	0 (0%)	3 (75%)
T2/FLAIR hyperintensity follow-up MRI	20 (22%)	3 (7.7%)	15 (37%)	0 (0%)	0 (0%)	2 (50%)
Mesial temporal T2/FLAIR hyperintensity follow-up MRI	17 (19%)	2 (5.1%)	14 (34%)	0 (0%)	0 (0%)	1 (25%)
Mesial temporal T2/FLAIR hyperintensity follow-up MRI, no HS	10 (11%)	0 (0%)	10 (24%)	0 (0%)	0 (0%)	0 (0%)

NMDAR, N-methyl-D-aspartate receptor; LGI1, leucine-rich glioma-inactivated 1, CASPR2, contactin-associated protein-like 2, GABAB, γ-aminobutyric acid B, AMPAR, α-amino-3-hydroxy-5-methyl-4-isoxazolepropinoic receptor; MRI, magnetic resonance imaging; FLAIR, fluid attenuated inversion recovery; HS, hippocampal sclerosis.

### Interrater assessments

Of 43 patients whose scans were evaluated by a second blinded neuroradiologist, 26 had a follow-up scan at least 3 months from the first scan available. In 25 of 26 cases, the follow-up MRI evaluated was the final scan for that patient. Interrater assessments are presented in Supplementary Table 3. An almost perfect inter-rater agreement was demonstrated for all variables.

### Initial radiological predictors of 12-month favourable mRS in NMDAR and LGI1 patients

**Figure 4:**
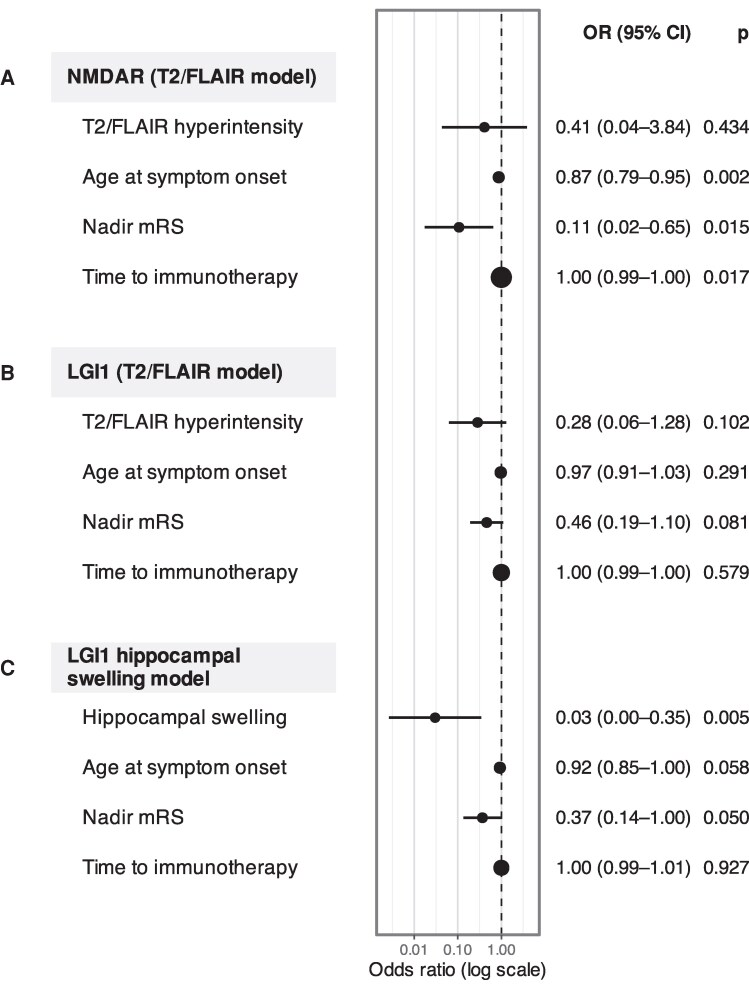
**Forest plots and corresponding multivariable logistic regression analyses for predictors of 12-month mRS.** (**A**) initial MRI T2/FLAIR hyperintensity anti-NMDAR (*n* = 51) and (**B**) anti-LGI1 Ab-mediated encephalitis (*n* = 50) models; (**C**) initial MRI hippocampal swelling anti-LGI1 Ab-mediated encephalitis (*n* = 50) model. Ab, antibody; CI, confidence interval; FLAIR, fluid-attenuated inversion recovery; LGI1, leucine-rich glioma-inactivated 1; mRS, modified Rankin scale; NMDAR, *N*-methyl-D-aspartate receptor; OR, odds ratio.

The presence T2/FLAIR hyperintensity on initial MRI was not associated with mRS status at 12 months in either NMDAR patients (OR 0.39; 95% CI 0.04, 3.97; *P* = 0.42) or LGI1 patients (OR 0.34; 95% CI 0.07, 1.57; *P* = 0.17) (Fig. 4 and Supplementary Table 4). In LGI1 patients, hippocampal swelling on initial MRI was associated with worse outcome, i.e. markedly reduced odds of favourable mRS at 12 months (OR 0.03; 95% CI 0.003, 0.34; *P* = 0.005) (Fig. 4). Adjusting for time to MRI did not considerably alter these findings (Supplementary Table 5). Data availability for regression models is illustrated in Supplementary Fig. 1.

### Initial radiological predictors of mesial temporal atrophy in LGI1 patients

**Figure 5:**
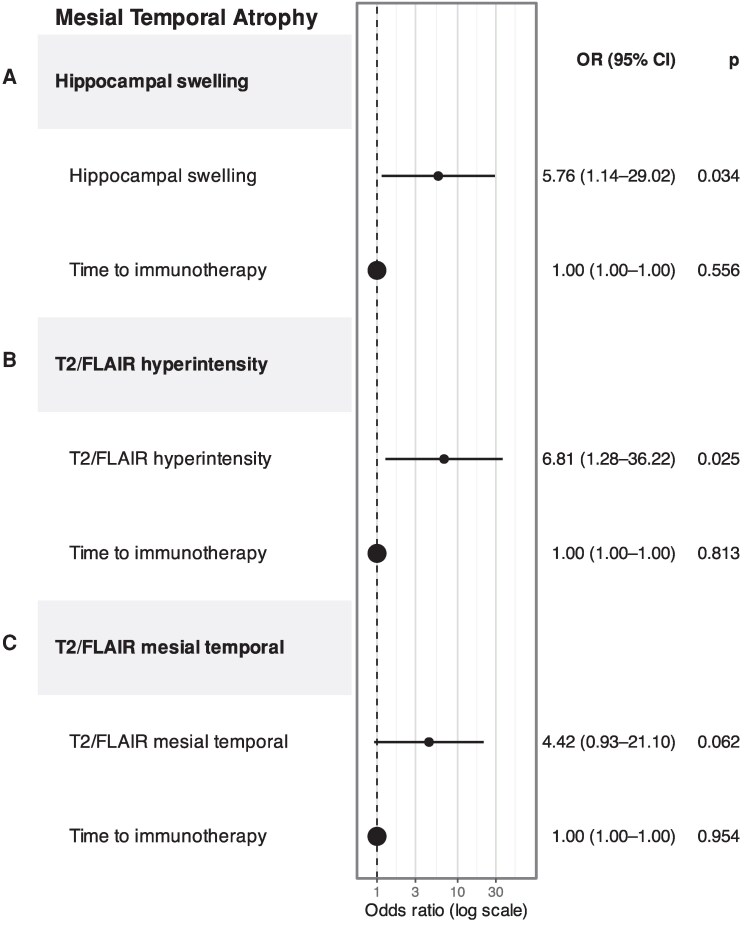
**Forest plots and corresponding multivariable logistic regression analyses for predictors of mesial temporal atrophy in LGI1 patients with follow-up MRI at least 3 months after initial (*n* = 35).** (**A**) Initial MRI hippocampal swelling model, (**B**) initial MRI T2/FLAIR hyperintensity model, and (**C**) initial MRI mesial temporal T2/FLAIR hyperintensity model for predictors of mesial temporal atrophy. Ab, antibody; CI, confidence interval; FLAIR, fluid-attenuated inversion recovery; LGI1, leucine-rich glioma-inactivated 1; OR, odds ratio.

In LGI1 patients, both hippocampal swelling (OR 5.76; 95% CI 1.14, 29.02; *P* = 0.03) and presence of any T2/FLAIR hyperintensity on initial MRI (OR 6.81; 95% CI 1.28, 36.22; *P* = 0.03) were associated with the development of mesial temporal atrophy on an MRI at least 3 months after the initial scan (Fig. 5 and Supplementary Table 6). Conversely, mesial temporal T2/FLAIR hyperintensity was not significantly associated with this outcome (OR 4.42; 95% CI 0.93, 21.10; *P* = 0.06).

### Initial radiological predictors of hippocampal sclerosis in LGI1 patients

**Figure 6:**
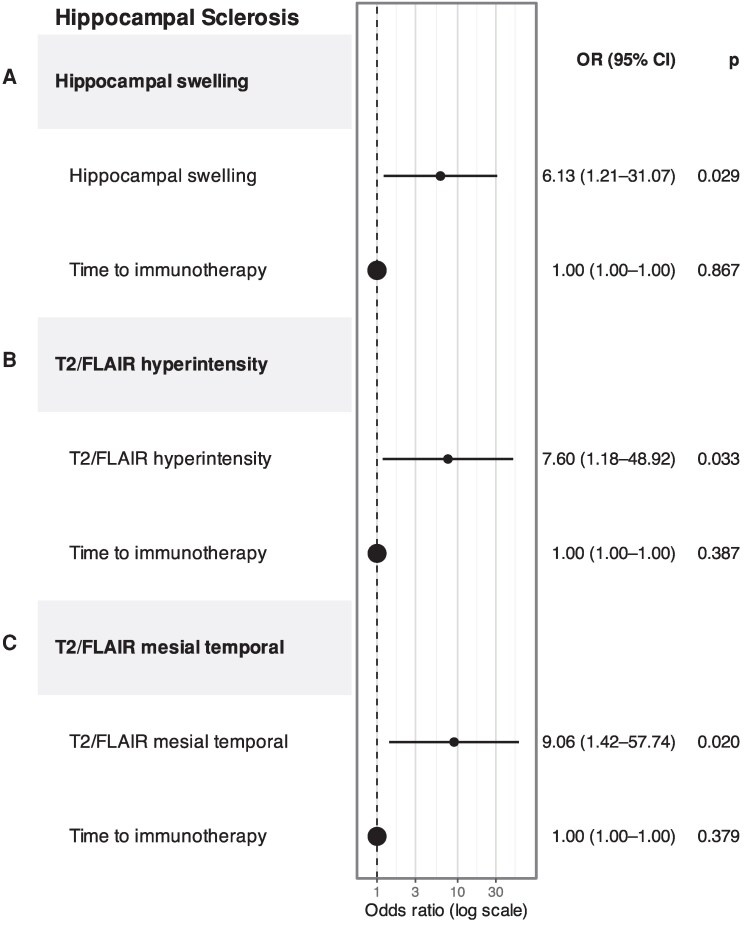
**Forest plots and corresponding multivariable logistic regression analyses for predictors of hippocampal sclerosis in LGI1 patients with follow-up MRI at least 3 months after initial (*n* = 35)**. (**A**) Initial MRI hippocampal swelling model, (**B**) initial MRI T2/FLAIR hyperintensity model, and (**C**) initial MRI mesial temporal T2/FLAIR hyperintensity model for predictors of mesial temporal atrophy. Ab, antibody; CI, confidence interval; FLAIR, fluid-attenuated inversion recovery; LGI1, leucine-rich glioma-inactivated 1; OR, odds ratio.

Development of hippocampal sclerosis was associated with hippocampal swelling (OR 6.13; 95% CI 1.21, 31.07; *P* = 0.03), and presence of any T2/FLAIR hyperintensity (OR 7.60; 95% CI 1.18, 48.92; *P* = 0.03), concordant with mesial temporal atrophy analyses (Fig. 6 and Supplementary Table 7). Additionally, initial mesial temporal T2/FLAIR hyperintensity was also associated with over 9-fold increased odds of developing hippocampal sclerosis (OR 9.06; 95% CI 1.42, 57.74; *P* = 0.02).

### Radiological predictors of significant memory impairment in LGI1 patients

We also assessed whether mesial temporal atrophy and hippocampal sclerosis on follow-up MRI was associated with the development of the clinically significant memory impairment in LGI1 patients. We found mesial temporal atrophy, adjusted for patient age, was associated with increased likelihood of significant memory impairment (OR 7.6; 95% CI 1.09, 52.84; *P* = 0.04), whereas hippocampal sclerosis was not significantly associated with same outcome (OR 5.38; 95% CI 0.87, 33.22; *P* = 0.07) (Table 4).

**Table 4:** Multivariable predictors of significant memory impairment at 12 months in patients with anti-LGI1 Ab-mediated encephalitis with a follow-up at least MRI 3 months after initial

Variable	OR	95% CI	*P*-value
*Mesial temporal atrophy model, n* = *31*			
** Mesial temporal atrophy**	**7**.**60**	**1.09, 52.84**	**0**.**04**
Age symptom onset	0.92	0.84, 1.00	0.06
*Hippocampal sclerosis model, n* = *31*			
Hippocampal sclerosis	5.38	0.87, 33.22	0.07
Age symptom onset	0.93	0.86, 1.01	0.07

Values in bold indicate statistical significance at the *P* < 0.05 level.

LGI1, leucine-rich glioma-inactivated 1.

## Discussion

In this study, we found a strong association between hippocampal swelling on initial brain MRI in LGI1 patients and poor functional outcome at 12 months. Conversely, initial T2/FLAIR hyperintensity in either LGI1 or NMDAR patients was not associated with worse functional outcome. In LGI1 patients, initial hippocampal swelling, hippocampal T2/FLAIR hyperintensity and mesial temporal hyperintensity were associated with later development of focal atrophy and/or hippocampal sclerosis. These radiological outcomes are important—development of mesial temporal atrophy correlated with persistent memory impairment at 12 months. MRI sequences other than T2/FLAIR were rarely abnormal in patients with Ab-mediated encephalitis, and a large proportion of NMDAR patients had normal initial MRI scans.

We observed hippocampal swelling in 22% of LGI1 patients, compared with mesial temporal T2/FLAIR hyperintensity in 35%. Few prior studies have described the incidence of acute hippocampal swelling, whereas several studies have assessed T2/FLAIR signal change. Commonly, the two coexist, but not always.^24^ In one study, 40% of patients had both swelling and increased T2 signal.^24^ Recently, hippocampal/amygdala swelling was described in 22% of patients with either anti-LGI1 or anti-CASPR2 Ab-mediated encephalitis.^25^ There are only three published cases of anti-LGI1 Ab-mediated encephalitis with neuropathology results. These cases all had hippocampal swelling. Despite this, the pathological correlate of acute hippocampal swelling at the cellular/molecular level is uncertain. Targeted brain biopsies from two cases prior to immunotherapy demonstrated activated astrocytes and either parenchymal or perivascular T-cell infiltration (including CD8^+^ T-cells), with neuronal complement deposition in one case.^26,27^ An autopsy case similarly revealed activated microglia and astrocytes, and CD8^+^ and CD48^+^ tissue infiltration with mild neuronal loss.^28^ Ultimately, our findings suggest a more severe disease course when acute hippocampal swelling is present, which may signify greater immune infiltration and microglial activation.

Hippocampi swelling were associated with a 97% reduction in the likelihood of a favourable mRS at 12 months. To our knowledge, prior studies have not evaluated the prognostic significance of such changes. Bilateral T2/FLAIR hyperintensity has been associated with long-term cognitive impairment,^10^ but an insufficient number of patients had this finding in our cohort to be able to confirm this. A previous retrospective study showed cognitive deficits during the initial disease stage and greater time to immunotherapy were linked to worse long-term functional outcomes.^4^ We found acute cognitive impairment occurred in almost all patients in our cohort. Therefore, we did not adjust our models for its presence or absence. We did, however, adjust relevant models for time to treatment. Acute hippocampal swelling may, therefore, signify a novel and readily assessable biomarker of disease severity in LGI1 patients and represent an opportunity for more intense immunotherapy early in the disease course to prevent worse outcomes. Future studies are warranted to confirm our finding.

In LGI1 patients, acute hippocampal swelling and T2/FLAIR hyperintensity were associated with an approximately 6-fold greater likelihood of focal atrophy on follow-up MRI. Almost all patients with T2/FLAIR hyperintensity on initial MRI had mesial temporal involvement. Therefore, it is likely that initial mesial temporal hyperintensity, rather than T2/FLAIR hyperintensity in general, is specifically related to mesial temporal atrophy and hippocampal sclerosis. In this study, the lack of significant association between mesial temporal signal and MRI sequelae may have been a consequence of the limited sample size. Hippocampal sclerosis is a known complication of anti-LGI1 Ab-mediated encephalitis and occurs in 44–48% of patients.^24,29^ It develops less commonly than mesial temporal atrophy.^20,24^ Additional implications, if any, of sclerosis compared with atrophy alone are unknown in anti-LGI1 Ab-mediated encephalitis. Notably, volume loss in the absence of initial signal change using volumetric imaging or high-resolution MRI with quantitative morphometry has also been previously reported.^30,31^

We found also that mesial temporal atrophy was associated with significant memory impairment, whereas hippocampal sclerosis was not. This discrepancy was likely due to the smaller number of patients with the latter and likely lack of sufficient power to better assess this relationship. Furthermore, hippocampal sclerosis diagnosis in this study may have been impacted by variable sensitivity of routine MRI based on availability of appropriate sequences, particularly coronal FLAIR of appropriate resolution.^18^ Additionally, it is possible that in limbic encephalitis, hippocampal sclerosis may be a final result, following hippocampal atrophy.^32^ If so, variability in follow-up scan timing could have contributed to the apparent discrepant relationship between 12-month memory impairment and atrophy or sclerosis. In practice, hippocampal atrophy without increased signal and sclerosis may be difficult to distinguish.^33^ Hippocampal subfield atrophy (with or without antecedent signal change) as quantified by neuroimaging software has been previously related to memory deficit on neuropsychology testing.^20,31^ Atrophy as determined by visual inspection may represent a more severe sequelae than as determined by volumetric or voxel-based techniques, but this has not been previously evaluated.

We found no association between initial T2/FLAIR hyperintensity and functional outcome at 12 months. In NMDAR patients, MRI abnormality was related to poor functional status at 12 months in one study^9^ but not so in others.^34^ Due to the low incidence of abnormalities in these patients a sample size larger than ours is required to investigate this effect. Notably, in both diseases, mRS is an insensitive measure of the burden of chronic morbidity.^35,36^ Therefore, the presence or extent of initial MR signal change may be best correlated with performance in neuropsychological assessment.

MRI abnormalities in NMDAR patients in our study were notably less frequent than previously reported.^3,9,12,37^ The median time to MRI in these patients was 17 (IQR 8,38) days in our cohort, consistent with the acute stage of the disease.^38^ Overall, 89% had their initial MRI prior to or on the day of first-line immunotherapy. MRI abnormalities are not part of proposed anti-NMDAR Ab-mediated encephalitis diagnostic criteria,^13^ though MRI is recommended when the diagnosis is considered. This includes patients presenting with first-episode psychosis.^39^ Our findings highlight the poor sensitivity of acute MRI for anti-NMDAR Ab-mediated encephalitis diagnosis and the importance of a broader panel of ancillary investigations in the workup, including CSF and EEG.

Abnormalities outside the mesial temporal lobes on T2/FLAIR sequences were infrequent in our cohort, and diffusion restriction and contrast enhancement were uncommon. These findings are consistent with recent studies.^8,25^ All patients with CEL and DWI positive lesions showed resolution of these findings on follow-up scans at variable intervals. In 4 of 6 patients the lesional T2/FLAIR signal persisted despite CEL or DWI resolution. Most of these patients presented with acute symptomatic seizures, though only one had SE. Acute MRI lesions in patients with SE are typically reversible,^21^ and are frequently DWI-positive, but less commonly show contrast enhancement.^21,40^ Patients with less frequent or shorter duration seizures may likely be scanned less urgently compared with those with SE, which limits these observations. Overall, while transient DWI-positive or CEL may be secondary to seizures in patients with AE, they can also directly reflect the underlying encephalitis. This is supported by the persistence of T2/FLAIR hyperintense signal after resolution of the DWI or CEL abnormalities in our cohort. Our findings also affirm the presence of diffusion restriction and contrast enhancement to be at least very rare in LGI1 patients, consistent with a prior study.^25^ Importantly, our findings suggest that persistence of either diffusion restriction or contrast enhancement should raise concern for an alternate diagnosis, such as an underlying glioma.

Limitations of our study include the largely retrospective nature of data collection, which impacted the availability and timing of follow-up MRI scans. Despite this, we could collate a relatively large cohort of patients with Ab-mediated encephalitis who had follow-up imaging available. Most patients in our cohort also had imaging performed prior to or on the day of immunotherapy, allowing a clearer depiction of the spectrum of abnormalities seen in acute disease. MRI protocol variability limited assessment of hippocampal sclerosis in some patients. Still, the subset of scans assessed by a second blinded neuroradiologist demonstrated almost-perfect inter-assessor agreement. This suggests that overall, the framework applied here may be generalizable despite scan protocol heterogeneity. For mesial temporal atrophy, hippocampal sclerosis and 12-month memory analyses, only patients with follow-up MRI scans were included, which may have introduced selection bias. Finally, retrospective assessment of memory dysfunction using the CASE does not capture objectively measured memory impairment and may not differentiate memory impairment from other cognitive domains such as attention as accurately as prospective neuropsychological assessments.

## Conclusion

Our study demonstrates that acute hippocampal swelling is associated with worse outcomes in LGI1 patients. Acute hippocampal swelling and encephalitis-associated T2/FLAIR hyperintensity are associated with subsequent development of focal atrophy and hippocampal sclerosis. Mesial temporal atrophy is associated with memory impairment in LGI1 patients. DWI-positive and CEL are uncommon in Ab-mediated encephalitis, but when present, are typically transient. Our findings expand on the diagnostic utility of MRI in Ab-mediated encephalitis and demonstrate the potential for prognostication in LGI1 patients.

## Supplementary Material

fcag193_Supplementary_Data

## Data Availability

Anonymized data is available to any qualified investigator by the corresponding author upon reasonable request and after approval from the relevant HREC. The R code used for statistical analysis and table generation is available at: https://github.com/nabilseery/Antibody-mediated-encephalitis-MRI-analysis-code.

## Appendix

Australian Autoimmune Encephalitis Consortium

Mastura Monif, Amy Brodtmann, Amy Halliday, Bruce Taylor, Cassie Nesbitt, Catherine Meade, Charles B Malpas, Chris Kyndt, Christina Kazzi, David Gillis, David Tarlinton, Darshi Ramanathan, Ernie G Butler, Foong Yi, Genevieve Skinner, Hannah Ford, Helmut Butzkueven, James Beharry, Jayashri Kulkarni, Jenny MacIntyre, Joshua Yap, Joanne Fielding, Kath Buzzard, Katherine Ko, Laurie McLaughlin, Marie O’Shea, Meaghan Clough, Meng Tan, Miri Forcadel, Nabil Seery, Nicola Warren, Noushin Chini Foroush, Owen White, Paul Beech, Richard A. Macdonell, Richard Wong, Robb Wesselingh, Rubina Alpitsis, Robert Bourke, Simon Broadley, Stefan Blum, Stephen W Reddel, Terence J O’Brien, Tiffany Rushen, Todd A Hardy, Tomas Kalincik, Tracie Tan, Udaya Seneviratne, Wen Wen Zhang and Wendyl J. D’Souza.
