# Supplementary material for: Acute and longitudinal magnetic resonance imaging abnormalities in antibody-mediated encephalitis
Source: Brain Commun. 2026 Jun 17;8(3):fcag193. doi: 10.1093/braincomms/fcag193 (PMC13273915; doi:10.1093/braincomms/fcag193)
Supplement: fcag193_Supplementary_Data [file fcag193_supplementary_data.docx]

**Supplementary material**

**Supplementary Table 1** – Cohort Demographics

| **Characteristic** | | **Overall, N = 137** | **NMDAR, N = 64** | **LGI1, N = 56** | **CASPR2, N = 10** | **GABAB, N = 2** | **AMPAR, N = 5** |
| --- | --- | --- | --- | --- | --- | --- | --- |
| Age onset | | 49 (27,66) | 27 (23,38) | 62 (53,72) | 65 (57,69) | 52 (44,60) | 56 (39,59) |
| Sex (male) | | 55 (40%) | 14 (22%) | 31 (55%) | 8 (80%) | 0 (0%) | 3 (60%) |
| Acute clinical features: | | | | | | | |
| Cognitive | | 127 (93%) | 58 (91%) | 52 (93%) | 10 (100%) | 2 (100%) | 5 (100%) |
| Seizures | | 101 (74%) | 40 (63%) | 51 (91%) | 7 (70%) | 2 (100%) | 1 (20%) |
| Status epilepticus | | 16 (12%) | 9 (14%) | 4 (7%) | 1 (10%) | 2 (100%) | 0 (0%) |
| Psychiatric | | 105 (77%) | 61 (95%) | 31 (55%) | 8 (80%) | 2 (100%) | 3 (60%) |
| Consciousness disturbance | | 47 (34%) | 35 (55%) | 8 (14%) | 2 (20%) | 2 (100%) | 0 (0%) |
| Speech disturbance | | 43 (31%) | 29 (45%) | 11 (20%) | 3 (30%) | 0 (0%) | 0 (0%) |
| Movement disorders | | 40 (29%) | 36 (56%) | 4 (7%) | 0 (0%) | 0 (0%) | 0 (0%) |
| Sleep disturbance | | 38 (28%) | 26 (41%) | 9 (16%) | 3 (30%) | 0 (0%) | 0 (0%) |
| First-line received | | 134 (98%) | 62 (97%) | 55 (98%) | 10 (100%) | 2 (100%) | 5 (100%) |
| 12m mRS | | 2 (1,2) | 2 (1,2) | 2 (1,2) | 2 (1,2) | 3 (2,3) | 2 (2,2) |
| 12m mRS favourable | | 88 (75%) | 39 (75%) | 38 (76%) | 7 (78%) | 1 (50%) | 3 (75%) |
| 12m CASE | | 2 (1,3) | 1 (1,3) | 2 (1,3) | 3 (2,3) | 1 (1,1) | 2 (2,2) |
| 12m significant  memory impairment | | 37 (33%) | 12 (24%) | 17 (35%) | 5 (63%) | 0 (0%) | 3 (75%) |
|  | | | | | |  |  |

NMDAR = N-methyl-D-aspartate receptor; LGI1 = leucine-rich glioma-inactivated 1; CASPR2 = contactin-associated protein-like 2; GABAB = γ-aminobutyric acid B; AMPAR = α-amino-3-hydroxy-5-methyl-4-isoxazolepropinoic receptor; mRS = modified Rankin scale; CASE = clinical assessment scale in autoimmune encephalitis

**Supplementary Table 2 –** Radiological Features of CEL and DWI Lesions

| Case | Diagnosis | CEL initial MRI, location | Diffusion restriction initial MRI, location | Accompanying T2/FLAIR hyperintense signal? | Follow-up MRI timing | CEL resolved? | Diffusion restriction resolved? | T2/FLAIR resolved? |
| --- | --- | --- | --- | --- | --- | --- | --- | --- |
| 1 | LGI1 | R) mesial temporal | No | Yes | 250 days | Yes | N/A | No |
| 2 | LGI1 | L) mesial temporal | No | Yes | 64 days | Yes | N/A | Yes |
| 3 | NMDAR | R) cerebellar hemisphere | No | Yes | 1567 days | Yes | N/A | Yes |
| 4 | AMPAR | R) mesial temporal | R) mesial temporal | Yes | 25 days | Yes | Yes | No |
| 4 | AMPAR | L) mesial temporal | No | Yes | 142 days | Yes | N/A | Yes |
| 5 | LGI1 | No | Bilateral mesial temporal | Yes | 108 days | N/A | Yes | No |
| 6 | LGI1 | No | L) basal ganglia | Yes | 35 days | N/A | Yes | No |

CEL = contrast enhancing lesion; DWI = diffusion weighted imaging; FLAIR = fluid attenuated inversion recovery; LGI1 = leucine-rich glioma-inactivated 1; AMPAR = α-amino-3-hydroxy-5-methyl-4-isoxazolepropinoic receptor

**Supplementary Table 3** – Interrater MRI Assessments

| Variable | Inter-assessor concordance^a,b,c^ | Gwet’s AC1 | p-value |
| --- | --- | --- | --- |
| *Initial MRI:* | | | |
| T2 hyperintensity | | | |
| Mesial temporal | 41/43 | 0.95 | 0 |
| Temporal | 43/43 | 1 | <0.001 |
| Extra-temporal | 41/43 | 0.95 | 0 |
| DWI lesion | 43/43 | 1 | 0 |
| CEL | 41/43 | 0.95 | 0 |
| Hippocampal size^d^ | 42/43 | 0.97 | 0 |
| *Follow-up MRI* |  |  |  |
| Hippocampal size^d^ | 24/26 | 0.91 | <0.001 |
| Hippocampal sclerosis | 23/26 | 0.87 | <0.001 |

CEL = contrast enhancing lesion; DWI = diffusion weighted imaging

^a^Agreement on presence or absence of T2/FLAIR hyperintensity for both mesial temporal regions

^b^Agreement on hippocampal size on both sides

^c^Agreement on presence or absence hippocampal sclerosis on both sides

^d^Hippocampal size either normal, swollen or atrophied

**Supplementary Table 4** – Univariable Logistic Regression for Predictors of Favourable mRS at 12 months in Patients with Anti-LGI1 Ab-Mediated Encephalitis and anti-NMDAR Ab-Mediated Encephalitis

| Variable | OR | 95% CI | p-value |
| --- | --- | --- | --- |
| *LGI1 (n = 50 for all models)* | | | |
| Age symptoms | 0.95 | 0.89, 1.00 | 0.07 |
| Sex (male) | 0.48 | 0.11, 2.12 | 0.33 |
| **Nadir mRS** | **0.42** | **0.21, 0.86** | **0.02** |
| Time to first-line immunotherapy | 1.00 | 1.00, 1.01 | 0.45 |
| **Hippocampal swelling** | **0.16** | **0.04, 0.77** | **0.02** |
| **T2/FLAIR hyperintensity initial** | **0.23** | **0.06, 0.92** | **0.04** |
| T2/FLAIR mesial temporal | 0.36 | 0.09, 1.37 | 0.13 |
| T2/FLAIR extra-mesial temporal | 0.30 | 0.02, 5.15 | 0.40 |
| *NMDAR (n = 51 for all models)* | | | |
| Age symptoms | **0.94** | **0.90, 0.98** | **0.009** |
| Sex (male) | 0.31 | 0.07, 1.25 | 0.10 |
| Nadir mRS | 0.53 | 0.24, 1.20 | 0.13 |
| Time to first-line immunotherapy | 1.00 | 1.00, 1.00 | 0.50 |
| T2/FLAIR hyperintensity initial | 0.74 | 0.12, 4.38 | 0.74 |

LGI1 = leucine-rich glioma-inactivated 1; NMDAR = N-methyl-D-aspartate receptor; mRS = modified Rankin scale; FLAIR = fluid attenuated inversion recovery

**Supplementary Table 5 –** Multivariable Evaluation of Initial T2/FLAIR Hyperintensity and Favourable mRS Status at 12 Months in Patients with Anti-NMDAR (*n=51*) Anti-LGI1 Ab-mediated Encephalitis, and Initial Hippocampal Swelling in Anti-LGI1 Ab-Mediated Encephalitis Adjusted for Time to MRI (*n= 50*)

| Variable | OR | 95% CI | p-value |
| --- | --- | --- | --- |
| *Anti-NMDAR Ab-mediated encephalitis (n=51)* | | | |
| **Age symptom onset** | **0.87** | **0.79, 0.95** | **0.002** |
| **Nadir mRS** | **0.13** | **0.02, 0.67** | **0.01** |
| **Time to MRI** | **1.00** | **0.99, 1.00** | **0.01** |
| T2/FLAIR hyperintensity on initial MRI | 0.34 | 0.04, 3.08 | 0.34 |
| *Anti-LGI1 Ab-mediated encephalitis T2/FLAIR model (n=50)* | | | |
| Age symptom onset | 0.97 | 0.91, 1.03 | 0.29 |
| Nadir mRS | 0.44 | 0.18, 1.08 | 0.07 |
| Time to first-line immunotherapy  Time to MRI | 1.00  1.00 | 0.99, 1.01  0.99, 1.01 | 0.42  0.57 |
| T2/FLAIR hyperintensity on initial MRI | 0.26 | 0.06, 1.23 | 0.09 |

*Anti-LGI1 Ab-mediated encephalitis hippocampal swelling (n=50)*

| **Age symptom onset** | **0.86** | **0.75, 0.99** | **0.03** |
| --- | --- | --- | --- |
| Nadir mRS | 0.39 | 0.15, 1.01 | 0.05 |
| Time to first-line immunotherapy Time to MRI | 0.99  1.02 | 0.98, 1.01  1.00, 1.05 | 0.25  0.08 |
| **Hippocampal swelling** | **0.02** | **0.002, 0.19** | **0.008** |

**Supplementary Table 6** - Univariable Predictors of Mesial Temporal Atrophy in Patients with Anti-LGI1 Ab-Mediated Encephalitis with a Follow-up MRI at Least 3 Months After Initial (n=35 for all models)

| Variable | OR | 95% CI | p-value |
| --- | --- | --- | --- |
| Age symptoms | 0.98 | 0.93, 1.04 | 0.53 |
| Sex (male) | 1.00 | 0.25, 3.92 | 1.00 |
| Nadir mRS | 0.55 | 0.24, 1.27 | 0.16 |
| Time to first-line immunotherapy | 1.00 | 1.00, 1.00 | 0.43 |
| **Hippocampal swelling** | **6.00** | **1.20, 30.01** | **0.03** |
| **T2/FLAIR hyperintensity** | **6.25** | **1.40, 27.93** | **0.02** |
| **T2/FLAIR mesial temporal hyperintensity** | **4.50** | **1.06, 19.11** | **0.04** |

LGI1 = leucine-rich glioma-inactivated 1; mRS = modified Rankin scale; FLAIR = fluid attenuated inversion recovery

**Supplementary Table 7** - Univariable Predictors of HS in Patients with Anti-LGI1 Ab-Mediated Encephalitis with a Follow-up at Least 3 months After Initial MRI (n=35 for all models)

| Variable | OR | 95% CI | p-value |
| --- | --- | --- | --- |
| Age symptoms | 0.98 | 0.92, 1.04 | 0.51 |
| Sex (male) | 0.86 | 0.20, 3.60 | 0.83 |
| Nadir mRS | 0.47 | 0.19, 1.21 | 0.12 |
| Time to first-line immunotherapy | 1.00 | 1.00, 1.00 | 0.88 |
| **Hippocampal swelling** | **6.00** | **1.21, 29.73** | **0.03** |
| **T2/FLAIR hyperintensity** | **5.33** | **1.10, 25.77** | **0.02** |
| **T2/FLAIR mesial temporal hyperintensity** | **6.48** | **1.32, 31.83** | **0.02** |

HS = hippocampal sclerosis; LGI1 = leucine-rich glioma-inactivated 1; mRS = modified Rankin scale; FLAIR = fluid attenuated inversion recovery

**Supplementary Figure 1 – Flow Diagram of Patient Inclusion and Data Availability.** Descriptions in brackets indicate outcome variable for relevant analysis. NMDAR patients, anti-*N*-methyl-D-aspartate receptor (NMDAR) antibody-mediated encephalitis patients; LGI1 patients, anti-leucine-rich glioma-inactivated 1 (LGI1) antibody-mediated encephalitis patients; mRS, modified Rankin scale; m, month.
